# Ethyl *N*-[3-(*N*,*N*-dimethyl­carbamo­yl)pyridin-2-ylsulfon­yl]carbamate

**DOI:** 10.1107/S160053681000663X

**Published:** 2010-02-27

**Authors:** Yan-Jun Hou, Wen-Yi Chu, Jun Sui, Zhi-Zhong Sun

**Affiliations:** a College of Chemistry and Materials Science, Heilongjiang University, Harbin 150080, People’s Republic of China

## Abstract

In the mol­ecular structure of the title compound, C_11_H_15_N_3_O_5_S, the amide group is nearly perpendicular to the pyridine ring, making a dihedral angle of 86.30 (13)°. The terminal ethyl group is disordered over two sites of equal occupancy. Inter­molecular N—H⋯O hydrogen bonding is present in the crystal structure.

## Related literature

The title compound is used in the preparation of nicosulfuron, a member of the sulfonyl­urea family of herbicides, see: Green & Ulrich (1993 ▶). For the synthesis, see: Murai *et al.* (1992 ▶).
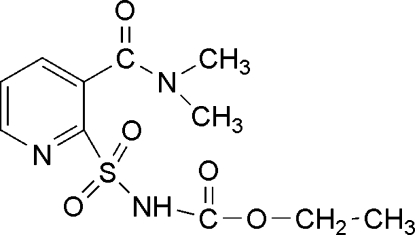


## Experimental

### 

#### Crystal data

C_11_H_15_N_3_O_5_S
*M* *_r_* = 301.32
Monoclinic, 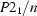

*a* = 8.4370 (11) Å
*b* = 11.1141 (15) Å
*c* = 15.074 (2) Å
β = 100.594 (2)°
*V* = 1389.4 (3) Å^3^
*Z* = 4
Mo *K*α radiation
μ = 0.26 mm^−1^
*T* = 296 K
0.17 × 0.16 × 0.15 mm

#### Data collection

Bruker SMART APEXII CCD area-detector diffractometer
Absorption correction: multi-scan (*SADABS*; Sheldrick, 1996 ▶) *T*_min_ = 0.958, *T*_max_ = 0.963
7979 measured reflections
3036 independent reflections
2384 reflections with *I* > 2σ(*I*)
*R*_int_ = 0.026

#### Refinement

*R*[*F* ^2^ > 2σ(*F* ^2^)] = 0.045
*wR*(*F* ^2^) = 0.129
*S* = 1.04
3036 reflections
195 parameters
H atoms treated by a mixture of independent and constrained refinement
Δρ_max_ = 0.29 e Å^−3^
Δρ_min_ = −0.47 e Å^−3^

### 

Data collection: *APEX2* (Bruker, 2004 ▶); cell refinement: *SAINT* (Bruker, 2004 ▶); data reduction: *SAINT*; program(s) used to solve structure: *SHELXS97* (Sheldrick, 2008 ▶); program(s) used to refine structure: *SHELXL97* (Sheldrick, 2008 ▶); molecular graphics: *SHELXTL* (Sheldrick, 2008 ▶); software used to prepare material for publication: *publCIF* (Westrip, 2010 ▶).

## Supplementary Material

Crystal structure: contains datablocks I, global. DOI: 10.1107/S160053681000663X/xu2723sup1.cif

Structure factors: contains datablocks I. DOI: 10.1107/S160053681000663X/xu2723Isup2.hkl

Additional supplementary materials:  crystallographic information; 3D view; checkCIF report

## Figures and Tables

**Table 1:** Hydrogen-bond geometry (Å, °)

*D*—H⋯*A*	*D*—H	H⋯*A*	*D*⋯*A*	*D*—H⋯*A*
N3—H3⋯O1^i^	0.87 (2)	1.91 (3)	2.773 (2)	172 (2)

Symmetry code: (i) 
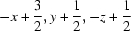
.

**Fig. 1.**
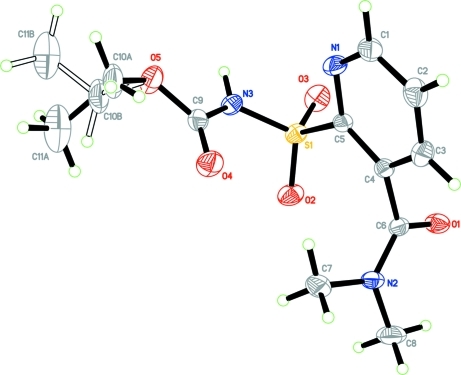
The molecular structure of the title compound, with displacement ellipsoids drawn at the 50% probability level. The disorder is shown.

**Fig. 2.**
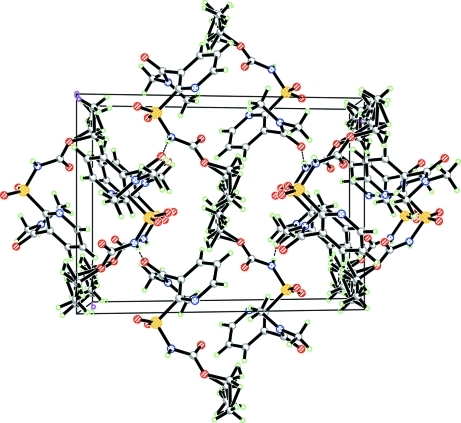
A part of packing of the crystal structure of the title compound, viewed down the a direction. Dashed lines indicate hydrogen bonds.

## supplementary crystallographic information

### Comment

The ethyl 3-(dimethylcarbamoyl)pyridin-2-ylsulfonylcarbamate is used for preparation of nicosulfuron, which is a member of the sulfonylurea family of herbicides (Green *et al.*, 1993).

The molecular structure is shown in Fig. 1. In the molecular structure the amide group is nearly perpendicular to the pyridine ring, the dihedral angle being 86.30 (13)°. Intermolecular N—H···O hydrogen bonding (Table 1) helps to stabilize the crystal structure.

### Experimental

To a solution of *N*,*N*-dimethyl-2-sulfamoylnicotinamide (10 mmol) and NaOH (12 mmol) in anhydrous toluene (50 ml) was added ethyl carbonochloridate (12 mmol). After stirring the mixture for 10 h at room temperature, the solvent was removed and 100 ml water was added. The oil after separation was concentrated under reduced pressure and the residue was recrystallized from methanol to give the title compound in a yield of 90% (Murai *et al.* 1992). Crystals suitable for single-crystal X-ray diffraction were obtained by recrystallization from ethanol at room temperature in a yield of 60%. Analysis found: C 43.9, H 4.9, N 13.9%; C_11_H_15_N_3_O_5_S requires: C 43.9, H 5.0, N 14.0%.

### Refinement

The ethyl group is disordered over two positions with 0.5 occupancy for each component. In the refinement. Imino H atom was located in a difference Fourier map and was refined isotropically. Other H atoms were placed in idealized positions with C—H = 0.96 (methyl), 0.97 (methylene) and 0.93 Å (aromatic), and refined in the riding-model approximation with U_iso_(H) = 1.5U_eq_(C) for methyl H atoms and 1.2U_eq_(C) for the others.

### Crystal data

**Table:**

C_11_H_15_N_3_O_5_S	*F*(000) = 632
*M**_r_* = 301.32	*D*_x_ = 1.441 Mg m^−^^3^
Monoclinic, *P*2_1_/*n*	Melting point = 436–437 K
Hall symbol: -P 2yn	Mo *K*α radiation, λ = 0.71073 Å
*a* = 8.4370 (11) Å	Cell parameters from 2785 reflections
*b* = 11.1141 (15) Å	θ = 2.3–26.9°
*c* = 15.074 (2) Å	µ = 0.26 mm^−^^1^
β = 100.594 (2)°	*T* = 296 K
*V* = 1389.4 (3) Å^3^	Prism, colorless
*Z* = 4	0.17 × 0.16 × 0.15 mm

### Data collection

**Table:**

Bruker SMART APEXII CCD area-detector diffractometer	3036 independent reflections
Radiation source: fine-focus sealed tube	2384 reflections with *I* > 2σ(*I*)
graphite	*R*_int_ = 0.026
φ and ω scans	θ_max_ = 27.1°, θ_min_ = 2.3°
Absorption correction: multi-scan (*SADABS*; Sheldrick, 1996)	*h* = −10→10
*T*_min_ = 0.958, *T*_max_ = 0.963	*k* = −13→14
7979 measured reflections	*l* = −19→18

### Refinement

**Table:**

Refinement on *F*^2^	Primary atom site location: structure-invariant direct methods
Least-squares matrix: full	Secondary atom site location: difference Fourier map
*R*[*F*^2^ > 2σ(*F*^2^)] = 0.045	Hydrogen site location: inferred from neighbouring sites
*wR*(*F*^2^) = 0.129	H atoms treated by a mixture of independent and constrained refinement
*S* = 1.04	*w* = 1/[σ^2^(*F*_o_^2^) + (0.0732*P*)^2^ + 0.3548*P*] where *P* = (*F*_o_^2^ + 2*F*_c_^2^)/3
3036 reflections	(Δ/σ)_max_ = 0.001
195 parameters	Δρ_max_ = 0.29 e Å^−^^3^
0 restraints	Δρ_min_ = −0.47 e Å^−^^3^

### Special details

**Table:**

Geometry. All esds (except the esd in the dihedral angle between two l.s. planes) are estimated using the full covariance matrix. The cell esds are taken into account individually in the estimation of esds in distances, angles and torsion angles; correlations between esds in cell parameters are only used when they are defined by crystal symmetry. An approximate (isotropic) treatment of cell esds is used for estimating esds involving l.s. planes.
Refinement. Refinement of *F*^2^ against ALL reflections. The weighted *R*-factor wR and goodness of fit *S* are based on *F*^2^, conventional *R*-factors *R* are based on *F*, with *F* set to zero for negative *F*^2^. The threshold expression of *F*^2^ > σ(*F*^2^) is used only for calculating *R*-factors(gt) etc. and is not relevant to the choice of reflections for refinement. *R*-factors based on *F*^2^ are statistically about twice as large as those based on *F*, and *R*- factors based on ALL data will be even larger.

### Fractional atomic coordinates and isotropic or equivalent isotropic displacement parameters (Å^2^)

**Table:**

	*x*	*y*	*z*	*U*_iso_*/*U*_eq_	Occ. (<1)
S1	0.81078 (6)	0.56615 (4)	0.24067 (3)	0.03700 (17)	
O1	1.05540 (17)	0.28321 (14)	0.28830 (10)	0.0475 (4)	
O2	0.97802 (19)	0.58254 (14)	0.27370 (11)	0.0526 (4)	
O3	0.7045 (2)	0.54186 (15)	0.30175 (11)	0.0554 (4)	
O4	0.90004 (19)	0.68423 (15)	0.08036 (11)	0.0539 (4)	
O5	0.6813 (2)	0.80460 (14)	0.06652 (12)	0.0552 (4)	
N1	0.6502 (2)	0.45464 (16)	0.10014 (12)	0.0432 (4)	
N2	1.19984 (19)	0.39369 (16)	0.20603 (12)	0.0407 (4)	
N3	0.7342 (2)	0.68519 (15)	0.18467 (12)	0.0379 (4)	
H3	0.641 (3)	0.709 (2)	0.1945 (16)	0.045 (6)*	
C1	0.6257 (3)	0.37404 (19)	0.03341 (15)	0.0480 (5)	
H1A	0.5289	0.3764	−0.0076	0.058*	
C2	0.7368 (3)	0.28809 (19)	0.02256 (15)	0.0490 (5)	
H2A	0.7162	0.2340	−0.0253	0.059*	
C3	0.8789 (3)	0.28309 (19)	0.08340 (15)	0.0434 (5)	
H3A	0.9543	0.2239	0.0778	0.052*	
C4	0.9110 (2)	0.36615 (16)	0.15349 (12)	0.0315 (4)	
C5	0.7912 (2)	0.45025 (16)	0.15719 (12)	0.0313 (4)	
C6	1.0625 (2)	0.34801 (17)	0.22302 (13)	0.0341 (4)	
C7	1.2086 (3)	0.4806 (2)	0.13467 (18)	0.0538 (6)	
H7A	1.1029	0.4929	0.0995	0.081*	
H7B	1.2790	0.4505	0.0965	0.081*	
H7C	1.2498	0.5555	0.1610	0.081*	
C8	1.3496 (3)	0.3661 (3)	0.26777 (18)	0.0624 (7)	
H8A	1.3293	0.3065	0.3105	0.094*	
H8B	1.3907	0.4378	0.2993	0.094*	
H8C	1.4273	0.3357	0.2342	0.094*	
C9	0.7842 (2)	0.72299 (17)	0.10698 (14)	0.0381 (4)	
C10A	0.698 (2)	0.8377 (11)	−0.0234 (15)	0.068 (3)	0.50
H10A	0.5943	0.8591	−0.0591	0.082*	0.50
H10B	0.7428	0.7715	−0.0528	0.082*	0.50
C11A	0.8134 (12)	0.9468 (6)	−0.0135 (5)	0.0974 (19)	0.50
H11A	0.8150	0.9806	−0.0720	0.146*	0.50
H11B	0.9201	0.9212	0.0133	0.146*	0.50
H11C	0.7768	1.0063	0.0242	0.146*	0.50
C10B	0.734 (2)	0.8705 (11)	−0.0110 (16)	0.068 (3)	0.50
H10C	0.8457	0.8953	0.0046	0.082*	0.50
H10D	0.7203	0.8205	−0.0646	0.082*	0.50
C11B	0.6287 (12)	0.9729 (6)	−0.0249 (5)	0.0974 (19)	0.50
H11D	0.6625	1.0259	−0.0682	0.146*	0.50
H11E	0.6330	1.0147	0.0312	0.146*	0.50
H11F	0.5203	0.9466	−0.0471	0.146*	0.50

### Atomic displacement parameters (Å^2^)

**Table:**

	*U*^11^	*U*^22^	*U*^33^	*U*^12^	*U*^13^	*U*^23^
S1	0.0368 (3)	0.0411 (3)	0.0317 (3)	0.0050 (2)	0.00264 (19)	−0.00259 (19)
O1	0.0327 (8)	0.0603 (9)	0.0480 (9)	−0.0012 (7)	0.0035 (6)	0.0214 (7)
O2	0.0417 (9)	0.0563 (9)	0.0520 (10)	0.0039 (7)	−0.0116 (7)	−0.0142 (7)
O3	0.0664 (11)	0.0635 (10)	0.0407 (9)	0.0091 (8)	0.0214 (8)	0.0056 (7)
O4	0.0500 (10)	0.0582 (10)	0.0589 (10)	0.0103 (8)	0.0243 (8)	0.0051 (8)
O5	0.0601 (11)	0.0493 (9)	0.0592 (10)	0.0162 (8)	0.0191 (8)	0.0155 (8)
N1	0.0331 (9)	0.0446 (10)	0.0461 (10)	0.0040 (7)	−0.0076 (7)	−0.0011 (8)
N2	0.0248 (8)	0.0466 (9)	0.0516 (10)	0.0009 (7)	0.0094 (7)	0.0087 (8)
N3	0.0364 (9)	0.0384 (9)	0.0401 (9)	0.0081 (7)	0.0098 (7)	−0.0021 (7)
C1	0.0455 (12)	0.0434 (12)	0.0472 (12)	−0.0058 (10)	−0.0125 (10)	−0.0005 (10)
C2	0.0632 (15)	0.0402 (11)	0.0406 (12)	−0.0055 (10)	0.0010 (10)	−0.0050 (9)
C3	0.0483 (12)	0.0385 (10)	0.0444 (11)	0.0060 (9)	0.0115 (10)	−0.0015 (9)
C4	0.0283 (9)	0.0337 (9)	0.0334 (9)	0.0000 (7)	0.0079 (7)	0.0058 (7)
C5	0.0280 (9)	0.0332 (9)	0.0316 (9)	−0.0002 (7)	0.0025 (7)	0.0013 (7)
C6	0.0274 (9)	0.0363 (9)	0.0390 (10)	0.0040 (7)	0.0071 (8)	0.0045 (8)
C7	0.0412 (13)	0.0582 (14)	0.0667 (16)	−0.0022 (10)	0.0219 (11)	0.0172 (12)
C8	0.0280 (11)	0.0805 (18)	0.0759 (17)	0.0020 (11)	0.0024 (11)	0.0118 (14)
C9	0.0383 (11)	0.0310 (9)	0.0453 (11)	−0.0002 (8)	0.0087 (9)	−0.0025 (8)
C10A	0.083 (7)	0.044 (6)	0.083 (6)	0.002 (4)	0.030 (5)	0.026 (6)
C11A	0.170 (6)	0.059 (2)	0.069 (3)	0.005 (3)	0.036 (4)	0.007 (2)
C10B	0.083 (7)	0.044 (6)	0.083 (6)	0.002 (4)	0.030 (5)	0.026 (6)
C11B	0.170 (6)	0.059 (2)	0.069 (3)	0.005 (3)	0.036 (4)	0.007 (2)

### Geometric parameters (Å, °)

**Table:**

S1—O2	1.4191 (16)	C3—H3A	0.9300
S1—O3	1.4241 (16)	C4—C5	1.385 (3)
S1—N3	1.6369 (18)	C4—C6	1.510 (3)
S1—C5	1.7871 (19)	C7—H7A	0.9600
O1—C6	1.230 (2)	C7—H7B	0.9600
O4—C9	1.202 (2)	C7—H7C	0.9600
O5—C9	1.324 (2)	C8—H8A	0.9600
O5—C10A	1.44 (2)	C8—H8B	0.9600
O5—C10B	1.51 (2)	C8—H8C	0.9600
N1—C1	1.334 (3)	C10A—C11A	1.544 (11)
N1—C5	1.335 (2)	C10A—H10A	0.9700
N2—C6	1.332 (2)	C10A—H10B	0.9700
N2—C7	1.458 (3)	C11A—H11A	0.9600
N2—C8	1.457 (3)	C11A—H11B	0.9600
N3—C9	1.381 (3)	C11A—H11C	0.9600
N3—H3	0.87 (2)	C10B—C11B	1.434 (19)
C1—C2	1.369 (3)	C10B—H10C	0.9700
C1—H1A	0.9300	C10B—H10D	0.9700
C2—C3	1.370 (3)	C11B—H11D	0.9600
C2—H2A	0.9300	C11B—H11E	0.9600
C3—C4	1.392 (3)	C11B—H11F	0.9600
			
O2—S1—O3	120.07 (11)	N2—C7—H7A	109.5
O2—S1—N3	110.48 (10)	N2—C7—H7B	109.5
O3—S1—N3	104.54 (9)	H7A—C7—H7B	109.5
O2—S1—C5	107.25 (9)	N2—C7—H7C	109.5
O3—S1—C5	109.34 (10)	H7A—C7—H7C	109.5
N3—S1—C5	104.06 (9)	H7B—C7—H7C	109.5
C9—O5—C10A	116.1 (8)	N2—C8—H8A	109.5
C9—O5—C10B	115.3 (7)	N2—C8—H8B	109.5
C10A—O5—C10B	18.8 (10)	H8A—C8—H8B	109.5
C1—N1—C5	117.19 (18)	N2—C8—H8C	109.5
C6—N2—C7	123.95 (18)	H8A—C8—H8C	109.5
C6—N2—C8	118.64 (18)	H8B—C8—H8C	109.5
C7—N2—C8	117.07 (18)	O4—C9—O5	126.6 (2)
C9—N3—S1	122.01 (14)	O4—C9—N3	124.59 (19)
C9—N3—H3	118.8 (16)	O5—C9—N3	108.80 (17)
S1—N3—H3	116.1 (16)	O5—C10A—C11A	106.2 (12)
N1—C1—C2	123.0 (2)	O5—C10A—H10A	110.5
N1—C1—H1A	118.5	C11A—C10A—H10A	110.5
C2—C1—H1A	118.5	O5—C10A—H10B	110.5
C1—C2—C3	118.8 (2)	C11A—C10A—H10B	110.5
C1—C2—H2A	120.6	H10A—C10A—H10B	108.7
C3—C2—H2A	120.6	C11B—C10B—O5	103.6 (13)
C2—C3—C4	120.37 (19)	C11B—C10B—H10C	111.0
C2—C3—H3A	119.8	O5—C10B—H10C	111.0
C4—C3—H3A	119.8	C11B—C10B—H10D	111.0
C5—C4—C3	115.90 (18)	O5—C10B—H10D	111.0
C5—C4—C6	126.36 (17)	H10C—C10B—H10D	109.0
C3—C4—C6	117.39 (17)	C10B—C11B—H11D	109.5
N1—C5—C4	124.70 (17)	C10B—C11B—H11E	109.5
N1—C5—S1	112.53 (14)	H11D—C11B—H11E	109.5
C4—C5—S1	122.77 (14)	C10B—C11B—H11F	109.5
O1—C6—N2	123.32 (18)	H11D—C11B—H11F	109.5
O1—C6—C4	118.27 (16)	H11E—C11B—H11F	109.5
N2—C6—C4	118.10 (17)		
			
O2—S1—N3—C9	62.57 (18)	N3—S1—C5—C4	138.92 (16)
O3—S1—N3—C9	−166.94 (17)	C7—N2—C6—O1	−173.2 (2)
C5—S1—N3—C9	−52.26 (18)	C8—N2—C6—O1	−0.1 (3)
C5—N1—C1—C2	0.9 (3)	C7—N2—C6—C4	13.3 (3)
N1—C1—C2—C3	0.9 (4)	C8—N2—C6—C4	−173.57 (19)
C1—C2—C3—C4	−1.7 (3)	C5—C4—C6—O1	85.0 (3)
C2—C3—C4—C5	0.7 (3)	C3—C4—C6—O1	−87.8 (2)
C2—C3—C4—C6	174.29 (19)	C5—C4—C6—N2	−101.2 (2)
C1—N1—C5—C4	−2.0 (3)	C3—C4—C6—N2	86.0 (2)
C1—N1—C5—S1	177.97 (16)	C10A—O5—C9—O4	8.8 (6)
C3—C4—C5—N1	1.2 (3)	C10B—O5—C9—O4	−12.0 (7)
C6—C4—C5—N1	−171.70 (18)	C10A—O5—C9—N3	−169.1 (6)
C3—C4—C5—S1	−178.77 (14)	C10B—O5—C9—N3	170.0 (7)
C6—C4—C5—S1	8.3 (3)	S1—N3—C9—O4	−10.6 (3)
O2—S1—C5—N1	−158.18 (15)	S1—N3—C9—O5	167.38 (14)
O3—S1—C5—N1	70.14 (16)	C9—O5—C10A—C11A	−91.5 (10)
N3—S1—C5—N1	−41.09 (16)	C10B—O5—C10A—C11A	1(4)
O2—S1—C5—C4	21.83 (18)	C9—O5—C10B—C11B	−164.2 (7)
O3—S1—C5—C4	−109.86 (17)	C10A—O5—C10B—C11B	99 (5)

### Hydrogen-bond geometry (Å, °)

**Table:**

*D*—H···*A*	*D*—H	H···*A*	*D*···*A*	*D*—H···*A*
N3—H3···O1^i^	0.87 (2)	1.91 (3)	2.773 (2)	172 (2)

Symmetry codes: (i) −*x*+3/2, *y*+1/2, −*z*+1/2.
